# *BRAF* 1799T>A Mutation Frequency in Mexican Mestizo Patients with Papillary Thyroid Cancer

**DOI:** 10.1155/2018/2582179

**Published:** 2018-04-02

**Authors:** Fernando Fernández-Ramírez, Luis M. Hurtado-López, Mario A. López, Eva Martínez-Peñafiel, Norma E. Herrera-González, Luis Kameyama, Omar Sepúlveda-Robles

**Affiliations:** ^1^Unidad de Genética, Hospital General de México, Dr. Balmis 148, Cuauhtémoc, 06720 México City, Mexico; ^2^Servicio de Cirugía, Hospital General de México, Dr. Balmis 148, Cuauhtémoc, 06720 México City, Mexico; ^3^Departamento de Genética y Biología Molecular, Centro de Investigación y de Estudios Avanzados del IPN, Av. Instituto Politécnico Nacional 2508, San Pedro Zacatenco, 07360 México City, Mexico; ^4^Sección de Estudios de Posgrado e Investigación, Escuela Superior de Medicina-IPN, Plan de San Luis y Díaz Mirón s/n, Miguel Hidalgo, 11340 México City, Mexico; ^5^Catedrático CONACyT, Unidad de Investigación Médica en Epidemiología Clínica, Hospital de Pediatría, Centro Médico Nacional Siglo XXI, IMSS, Av. Cuauhtémoc 330, Cuauhtémoc, 06270 México City, Mexico

## Abstract

Thyroid cancer is the most frequent endocrine malignancy, and its incidence and prevalence are increasing worldwide. Despite its generally good prognosis, the observed mortality rates are higher in the less-developed regions. This indicates that timely diagnosis and appropriate initial management of this disease are important to achieve a positive outcome. We performed an observational study in order to describe the frequency of the* BRAF* 1799T>A mutation in Mexican mestizo patients with thyroid nodules, a scarcely studied ethnic group with large populations. Competitive allele-specific Taqman PCR was performed in 147 samples of thyroid tissue DNA obtained from patients histologically diagnosed with papillary thyroid cancer (PTC), colloid goiters, and follicular adenomas. The* BRAF* 1799T>A mutation frequency was 61.1% in PTC samples (*p* = 4.99 × 10^−11^). Potential diagnostic values were as follows: sensitivity, 61.1%; specificity, 96%; PPV, 94.2%; NPV, 69.5%; accuracy, 77.9%. Taking into account the fact that this mutation is not frequently found in cytologically indeterminate nodules, we suggest that the* BRAF* mutational analysis should be implemented in the clinical setting along with other diagnostic criteria such as USG, in order to contribute to diagnosis and to surgical decision-making during the initial management of thyroid nodules in Mexican public hospitals.

## 1. Introduction

The prevalence of palpable thyroid nodules in Mexico City, which has a population over 20 million, is 1.4%. The majority of these nodules correspond to colloid goiters (CG, 47.2%), followed by follicular adenomas (FA, 23.5%), Hashimoto's thyroiditis (20.5%), and papillary carcinomas (PTC, 5.9%), the latter of which constitute the most frequent endocrine malignancy [1]. Despite the fact that the basis for thyroid nodule presurgical diagnosis is the cytological examination of fine-needle aspiration biopsies (FNAB), approximately 30% of these analyses generate inconclusive reports [2, 3]. Consequently, the patients with undetermined or nondiagnostic FNAB must undergo diagnostic surgery, in which the resected tissue is analyzed by the histological gold standard.

Molecular analysis of thyroid nodules has been widely proposed as a preliminary assessment to reduce the number of unnecessary diagnostic surgeries. Specifically, it has been shown that testing for* BRAF* mutations contributes to the diagnosis of thyroid cancer, even at early stages [4, 5]. The 1799T>A transversion in gene* BRAF *is the most common mutation found in PTC, with a global mean of 50%. However, its frequency is widely variable due to the genetic and environmental background of the patients, as well as the clinical characteristics of the tumors. For instance, in carcinomas measuring ≤1.0 cm in size, the frequency of BRAF V600E ranges from 17% to 77% among different populations [6]. Therefore, its diagnostic value would differ in geographical areas with different prevalence rates of the mutation [2]. In addition, the observed frequency of this mutation in the inconclusive FNAB cytopathological categories is low, ranging from 2.7% to 39.1% in lesions of undetermined diagnosis (atypia/follicular lesion of undetermined significance, AUS/FLUS) [4, 5, 7, 8] and from 30.7% to 37% in the suspicious categories [4, 5], which include follicular neoplasm, suspicious for a follicular neoplasm, and suspicious for malignancy (FN/SFN/SM) [9].

Several meta-analyses indicate that* BRAF* 1799T>A is associated with aggressive clinicopathological features of PTC, but its use as a prognostic marker is a current subject of debate due to contrasting results [10]. Taking these considerations into account, the utility of* BRAF* testing of thyroid nodules must therefore be explored in specific populations [5]. In this observational study, we describe the frequency of the* BRAF* 1799T>A mutation in resected thyroid tumor DNA samples from Mexican mestizo patients, to make a preliminary assessment of its potential diagnostic utility in such population.

## 2. Materials and Methods

### 2.1. Patients and Samples

This study included 147 tissue samples from consecutive euthyroid patients living in Mexico City, who underwent total thyroidectomy for PTC or diagnostic surgery for thyroid nodules at the General Hospital of Mexico, from September 2010 to December 2011. All of the patients signed an informed consent document approved by the Ethics Board of the hospital. Approximately 20 mg tumor samples were taken from the surgically resected tissues and, when available, paired healthy thyroid tissue samples were obtained from the position most distal to the nodule (contralateral lobe). Samples were classified by their definitive diagnosis, which was obtained from histological analyses (gold standard). No presurgical FNAB cytological data was incorporated into this study, as it was unavailable. DNA was purified using the Axyprep Multisource Genomic DNA Miniprep kit (Corning Inc., NY, USA).

### 2.2. Competitive Allele-Specific Taqman PCR (castPCR)

We used castPCR to selectively amplify mutant alleles in the tumor DNA samples, as a positive/negative presence assay. Mutation detection reactions were prepared with Taqman assays BRAF_476_mut (Cat. #4465804) and BRAF_rf (Cat. #4465807), following the manufacturer's protocol. All tests were performed at least by duplicate in a StepOne Plus thermal cycler, and the Mutation Detector software was used for analysis, assuming a 1% minimal detection cutoff. All of the materials, instruments, and tools used for castPCR analysis were from Thermo Fisher Scientific Inc. (Waltham, MA, USA).

### 2.3. Statistical Analysis

The chi square with Yates' continuity correction was employed to test the association of the* BRAF* mutation with any of the studied tissue types, and Fisher's exact test was used for pairwise comparisons. The distribution of age and gender among the histological groups was compared by Kruskal–Wallis' and Yates' tests, respectively. Diagnostic and predictive values were calculated as previously reported [2, 11, 12]. The association of the clinical variables with PTC recurrence was explored by binary logistic regression using SPSS statistics 20.0 (IBM Corp.), including as clinical covariates the age and gender of the patient, the PTC histological subtype,* BRAF* 1799T>A mutational status, TNM stage as well as individual primary tumor (T), regional nodes (N) and distant metastasis (M) classification values, ultrasonographic (USG) classification of nodules according to the Thyroid Imaging Reporting and Data System [13], tumor size (length) according to USG, and the presence of vascular and capsular invasion (*n* = 39). A *p* < 0.05 was considered significant for all analyses.

## 3. Results

A total of 147 samples were collected, and these corresponded to PTC, CG, and FA, according to histological gold standard (Table 1). Six of the PTC cases corresponded to the follicular variant, and 43 samples of healthy thyroid tissues from PTC patients (HT-PTC) were included in the screening as well. The* BRAF* 1799T>A mutation frequency in PTC samples was 61.1%. Taken together, our data indicate that this mutation is significantly associated with malignancy (Yates'* p *= 4.99 × 10^−11^) and constitutes a risk factor when compared to grouped benign diagnoses (Table 1, total benign). Conversely, no association was found with benign lesions neither with HT-PTC, and this was consistent in paired comparisons (Table 1). Within a 5-year follow-up, 6 out of the 54 PTC cases presented relapse, which was significantly associated with the initial TNM classification of the tumors (*p* = 0.014), regardless of the presence of the* BRAF* mutation (Table 2). Moreover, 3 out of 43 HT-PTC samples were positive for the* BRAF* 1799T>A mutation (Table 1), but none of the corresponding patients exhibited recurrence in the same period of time. Quantification of mutant alleles in 20 positive PTC samples revealed a wide variation among tumors, ranging from 4 ± 1% to 63 ± 13% (mean = 39.2 ± 18.7%, median = 41.4%).

## 4. Discussion

The incidence and prevalence of thyroid cancer are increasing worldwide, and the mortality rates are higher in the less-developed regions [14, http://globocan.iarc.fr]. This indicates that, despite the fact that thyroid cancer has a good prognosis in general terms, its diagnosis and management are still important to achieve a positive outcome. The clinical importance of thyroid nodules lies in the need to exclude thyroid cancer [15], which occurs in 7–15% depending on individual, genetic, and environmental factors [16, 17]. Therefore, the use of molecular approaches has become a fundamental aspect to improve thyroid nodule management. In addition, presurgical BRAF V600E mutation testing may guide in defining the optimal extent of initial thyroid surgery [2, 18].

The frequency of the* BRAF* 1799T>A mutation in Mexican PTC samples was 61.1%, which is higher than the estimated global mean frequency [6, 10]. The potential diagnostic values were as follows: sensitivity, 61.1%; specificity, 96%; PPV, 94.2%; NPV, 69.5%; accuracy, 77.9%. This mutation constitutes a risk factor for malignancy (OR = 37.7, CI = 8.2–171.8). While these data are comparable to those reported with other molecular methods, a high PPV but low sensitivity and NPV values were observed. In addition, the* BRAF* 1799T>A substitution is not frequently found in thyroid nodules with indeterminate FNAB cytology [4, 5, 7, 8]. Therefore,* BRAF* molecular testing per se may not be as useful in these cases. In general terms, the diagnostic performance of* BRAF* testing improves when it is combined with conventional diagnostic methods, even in studies without false-positives [2, 19, 20]. In nodules with suspicious cytology (i.e., the Bethesda categories IV and V [9]), mutational testing for BRAF may be considered after clinical and USG features, if such data would be expected to affect surgical decision-making [15].

In our studied cohort, 11% of the PTC cases presented relapse within 5 years, and the death rate was 1.8%. These data are in the range reported in previous studies [21, 22]. Despite the fact that the* BRAF* mutational status was not significantly associated with PTC recurrence (*p* = 0.11, Table 2), studies in larger cohorts are warranted to determine its potential prognostic utility in Hispanic populations. The quantification of the mutant alleles in the samples confirmed the clonal heterogeneity of PTC, supporting the reconsideration of BRAF V600E as a secondary mutation rather than an activating genetic event [10, 23]. However, the different mechanisms of tumor activation and clonal progression in PTC are still unknown and will demand further study.

## 5. Conclusions

The frequency of the* BRAF* 1799T>A mutation in the Mexican PTC cases is 61.1%. Considering its low sensitivity and NPV values, in addition to its low frequency in indeterminate nodules, the* BRAF* mutational testing could only be useful if implemented in combination with other diagnostic criteria, such as ultrasonography, in order to contribute to diagnosis and surgical decision-making during the initial management of thyroid nodules in Mexican hospitals.

## Figures and Tables

**Table 1:** Frequency of the *BRAF* 1799T>A mutation in the included thyroid tissue samples.

Diagnostic group	Number of samples	Mutation-positive samples	*p* value^*∗*^	OR (CI)
PTC	54	33 (61.1%)	-	-
CG	39	1 (2.5%)	7.2 × 10^−10^	59.7 (7.6–468)
FA	11	1 (9.1%)	0.0016	15.7 (1.8–131)
Total benign	50	2 (4.0%)	1 × 10^−10^	37.7 (8.2–171)
HT-PTC	43	3 (6.95%)	1.3 × 10^−8^	NA

Samples correspond to histologically diagnosed papillary thyroid cancer (PTC), colloid goiter (CG), follicular adenoma (FA), and healthy thyroid tissues from PTC patients (HT-PTC). Total benign represents the sum of CG and FA cases. ^*∗*^The *p* value refers to the positive association of the *BRAF* mutation with PTC, as compared to each diagnosis by Fisher's exact test. Likewise, the odds ratio (OR) and 95% confidence interval (CI) were also calculated. NA = not applicable. The distribution of age (*p* = 0.15) and gender (*p* = 0.89) among diagnostic groups displayed no significant differences.

**Table 2:** Clinical characteristics of the studied patients.

Clinical variable	Details (*n*)	*p* value^*∗*^
Age	44.2 ± 13.5 (mean ± SD)	0.744

Gender	Male = 7	0.947
	Female = 32	

PTC subtype	PTC = 33	0.057
	FV-PTC = 6	

BRAF 1799T>A	Positive = 27	0.110
	Negative = 12	

TNM stage	I = 22	**0.014**
	II = 4	
	III = 5	
	IVa = 8	

TIRADS^*∗∗*^	2 = 12	0.760
	3 = 13	
	4a = 3	
	4b = 4	
	5 = 2	
	6 = 5	

Size (USG length)	3.4 ± 1.9 (mean ± SD)	0.825

Capsular invasion	Positive = 24	0.379
	Negative = 15	

Vascular invasion	Positive = 11	0.244
	Negative = 28	

Primary tumor (T)	T1 = 11	0.341
	T2 = 16	
	T3 = 9	
	T4a = 1	
	T4b = 2	

Regional nodes (N)	N0 = 12	0.896
	N1a = 11	
	N1b = 9	
	NX = 7	

Recurrence	Positive = 6	-
	Negative = 33	

Data was compiled from the clinical records. ^*∗*^The *p* value refers to the association of the clinical variable with PTC recurrence according to the logistic regression analysis (*n* = 39); significance (*p* value < 0.05) is indicated in bold font. ^*∗∗*^The USG classification of nodules was according to the Thyroid Imaging Reporting and Data System [9]. FV-PTC is the follicular variant of PTC. None of the samples displayed distant metastasis.
